# Supplementary material for: Qualitative similarities and differences in visual object representations between brains and deep networks
Source: Nat Commun. 2021 Mar 25;12:1872. doi: 10.1038/s41467-021-22078-3 (PMC7994307; doi:10.1038/s41467-021-22078-3)
Supplement: Supplementary file 1 — Supplementary Information [file 41467_2021_22078_MOESM1_ESM.pdf]

SUPPLEMENTARY MATERIAL

for

Qualitative similarities and differences in visual object representations between brains and deep networks

Georgin Jacob, RT Pramod, Harish Katti & SP Arun

CONTENTS

SECTION S1. GENERALIZATION ACROSS INSTANCES OF VGG-16

SECTION S2. GENERALIZATION ACROSS FEED FORWARD NETWORKS

SECTION S3. GENERALIZAITON ACROSS DISTANCE METRICS

SECTION S4. LAYER-WISE ANALYSIS OF SCENE INCONGRUENCE

SECTION S5. SUPPLEMENTARY REFERENCES

SECTION S1. GENERALIZATION ACROSS INSTANCES OF VGG-16

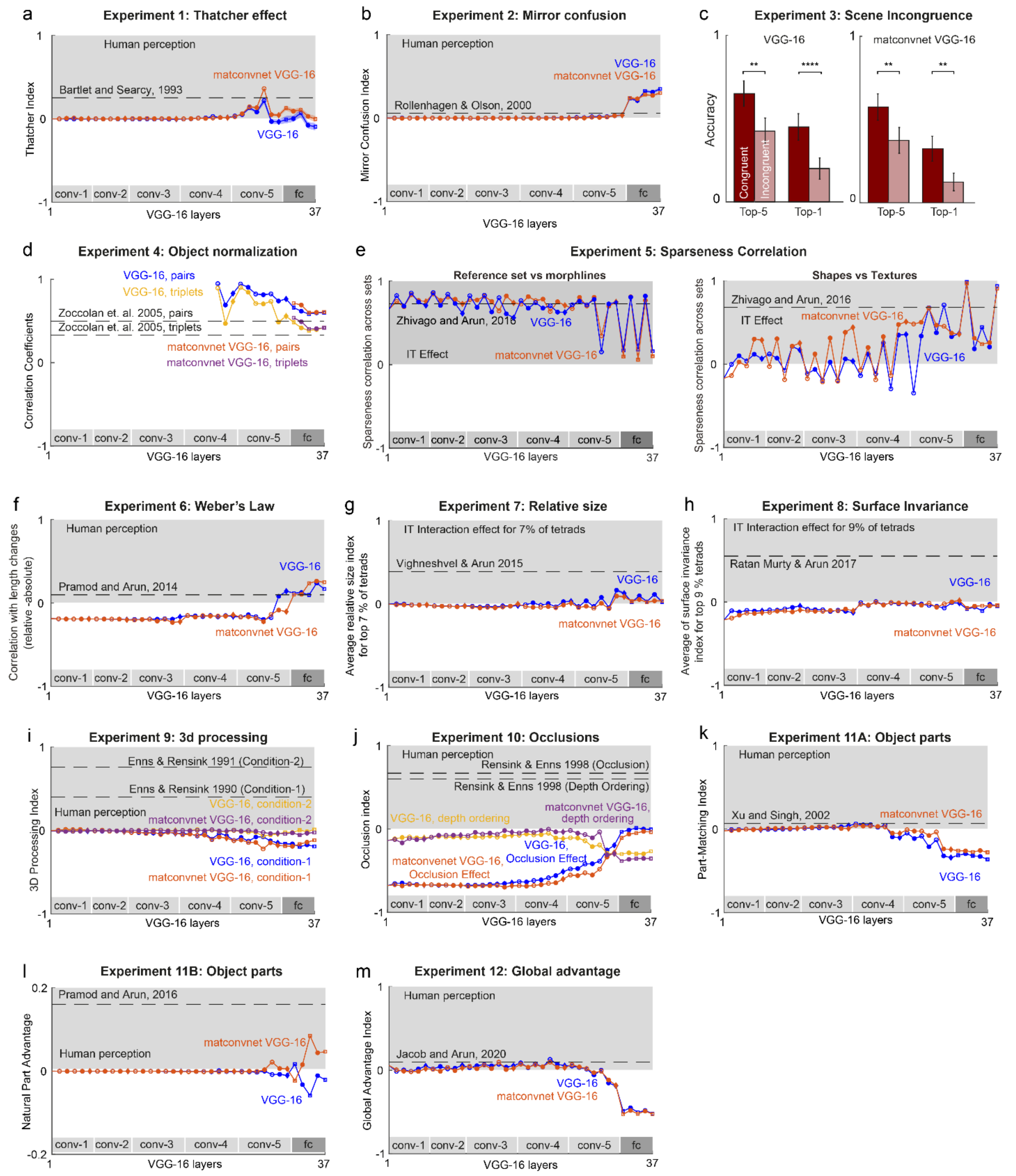

Figure S1. Results using another instance of the VGG-16 network. The results for each experiment are shown for both the MatConvNet VGG-16 (*red*) and VGG-16 (*blue*). The grey region indicates humanlike performance.

a) Thatcher index across layers of the VGG networks. Error bars indicate s.e.m across face pairs (n = 20). Dashed lines represent the Thatcher index from a previous study [1], measured on a different set of faces (see Methods).
b) Mirror Confusion Index (averaged across all objects) across layers for the pretrained VGG-16 networks. Error bars indicates s.e.m across stimuli (n = 50). Dashed lines represent the mirror confusion index estimated from monkey inferior temporal (IT) neurons in a previous study [2].
c) Accuracy of object classification by the VGG-16 network for congruent (*dark*) and incongruent (*light*) scenes, for top-5 accuracy (*left*) and top-1 accuracy (*right*). Error bars represent s.e.m across all scene pairs (n = 40). Asterisks indicate statistical significance computed using the Binomial probability of obtaining a value smaller than the incongruent scene correct counts given a Binomial distribution with the congruent scene accuracy (** is $p < 0.01$, **** is $p <0.001$).
d) Normalization slope plotted across layers for two object displays (*blue & red*) and three-object displays (*yellow & purple*) for the VGG-16 network and MatConvNet VGG-16. The dashed lines depict the slopes observed in monkey IT neurons using a different stimulus set [3].
e) *Left:* Correlation between sparseness on the reference set vs along morphlines across units of each layer in VGG-16 networks. The dashed line indicates the observed correlation in monkey IT neurons on the same set of stimuli. *Right:* Correlation between sparseness for textures and sparseness for shapes plotted across layers of the VGG-16 networks. The dashed line indicates the observed correlation in monkey IT neurons on the same set of stimuli.
f) To calculate a single quantity that measures adherence to Weber's law, we calculated the correlation between distances and relative changes in length and subtracted the correlation between distances with absolute changes in length (see Methods). A positive difference indicates adherence to Weber's law (*gray region*). This difference in correlation is plotted across layers for line length in the VGG-16 networks. The dashed line indicates the value observed during human performing visual search on the same stimuli.
g) Relative size index across units with interaction effects (averaged across top 7% tetrads, error bars representing s.e.m) across layers of the VGG-16 networks. The dashed line shows the strength of the relative size index estimated from monkey IT neurons on the same set of stimuli [4].
h) Surface invariance index across units with interaction effects (averaged across top 9% pattern/surface tetrads, error bars representing s.e.m) across layers of the VGG-16 networks. The dashed line depicts the surface invariance index estimated from monkey inferior temporal neurons on the same set of stimuli [5].
i) 3D processing index for the VGG-16 networks across layers, for condition 1 and condition 2 (Figure 5A). Dashed lines represent the estimated human effect measured using visual search on the same stimuli.
j) Occlusion index for the occlusion and depth ordering effects for each layer of VGG-16 networks. Dashed lines represent the effect size in humans measured using visual search on the same stimuli.

k) Part processing index across layers of the VGG-16 networks. The dashed line represents the effect size estimated from human visual search on the same stimuli [6].

l) Natural part advantage across layers of the VGG-16 networks. The dashed line represents the effect size estimated from human visual search on the same stimuli [7].

m) Global Advantage index across layers of the VGG-16 networks. The dashed line represents the effect size estimated from human visual search on the same stimuli [8].

SECTION S2. GENERALIZATION ACROSS FEED FORWARD NETWORKS

The results in the main text were based on testing a specific feedforward network, namely VGG-16. Here, we investigated other feedforward network architectures for the presence of the same perceptual and neural phenomena. We did not test the recurrent networks since unfolding recurrent networks over time make them equivalent to a deep feedforward network [9,10].

Methods

We selected four popular pre-trained feedforward networks, all trained on the ImageNet ILSVRC challenge data [11,12]. We selected architectures that are shallower and deeper than VGG-16, to investigate whether the depth of the network influences the emergence of the perceptual and neural properties. All networks were implemented using MatConvNet framework in MATLAB, and their performance is summarized in Table S1.

Network 1: AlexNet. This network won the ILSVRC 2012 challenge by a large margin [13]. The network consists of five convolutional layers and three fully-connected layers. Drop-out technique is used fully connected layers to reduce overfitting. The architecture of this network is shallower compared to VGG-16.

Network 2: GoogLeNet. This network follows the inception architecture which is well known for better utilization of computing resources inside the network. This network won the classification track of the ILSVRC 2014 challenge [14].

Network 3: ResNet-50. ResNet-50 is a shallower variant of the ResNet-152 detailed below.

Network 4: ResNet-152. The network uses a residual learning principle which make them capable of training deeper networks without the problem of vanishing gradients [15]. The ResNet architecture won three tracks (classification, detection and localization) of the ILSVRC 2015 challenge and two tracks (detection and segmentation) of the COCO 2015 challenge.

**Name of network**	**Performance**	
	**Top-1 error (%)**	**Top-5 error (%)**
AlexNet	42.6	19.6
VGG-16	28.5	9.9
VGG-16 trained using MatConvNet	28.3	9.5
GoogLeNet	34.2	12.9
ResNet-50	24.6	7.7
ResNet-152	23.0	6.7

Table S1. Performance of deep networks on the ILSVRC 2012 validation dataset (accuracy reported from MatConvNet website, accessed on 27th November 2019).

Results

Experiment 1: Thatcher effect. The Thatcher index for each network across layers is shown in Figure S1A. It can be seen that the Thatcher index is negative for all networks in their final layers except for GoogLeNet which showed a small positive level in the final layers. For the networks with higher classification performance (GoogLeNet, ResNet-50, ResNet-152), we observed an interesting pattern whereby the Thatcher index is positive in the intermediate layers. This is true even for the VGG-16 network (Figure 2B).

Experiment 2: Mirror Confusion. The mirror confusion index for each network is shown in Figure S1B. All networks exhibited an increasing mirror confusion index across layers, just as we observed for VGG-16 (Figure 2D).

Experiment 3: Scene incongruence. The classification accuracy for objects in congruent and incongruent scenes is shown for each network in Figure S1C. It can be seen that the deeper architectures show smaller incongruence effects.

Experiment 4: Multiple object normalization. The normalization slope for pairs and triplets for all networks is shown in Figure S1D. It can be seen that there is increased normalization in the later layers in all networks.

Experiment 5: Selectivity across multiple dimensions. The correlation between sparseness of units in each layer for textures and shapes is shown in Figure S1E. It can be seen that all networks show an increasing trend in later layers. We obtained qualitatively similar results for comparing sparseness on the reference shape set and morph lines (not shown for brevity).

Experiment 6. Weber's law. The Weber's law measure (difference in correlation for relative vs absolute length) for all networks is shown in Figure S2A. It can be seen that the Weber's law arises in the later layers for all the networks.

Experiment 7. Relative size. The relative size effect for each network across layers is shown in Figure S2B. It can be seen that the relative size effect is extremely weak and variable across networks, and never approaches the levels observed in the brain (relative size index = 0.39).

Experiment 8: Decoupling patterns from surfaces. The surface invariance index for each network across layers is shown in Figure S2C. It can be seen that the index is consistently negative for all networks, as observed for VGG-16.

Experiment 9: 3D processing. The 3D processing indices (for Condition 1 & 2) for each network across layers is shown in Figure S2D. It can be seen that the 3D processing indices are generally negative for all networks, and even if the index is positive, the levels are much smaller than observed in humans.

Experiment 10: Occlusions. The occlusion indices for each network across layers is shown in Figure S2E. It can be seen that both indices are consistently negative across layers, as observed for VGG-16.

Experiment 11: Object parts. The natural part advantage for Experiment 11B is shown for each network across layers in Figure S3B. It can be seen that the natural part advantage is highly variable across networks, with GoogLeNet showing levels comparable to humans in the later layers.

Experiment 12: Global shape advantage. The global advantage index for each network across layers is shown in Figure S3C. Across all networks, there is a slight global advantage in the intermediate layers, which reverses into a local advantage in the later layers. Thus, it appears that all the feedforward networks are using local features for classification.

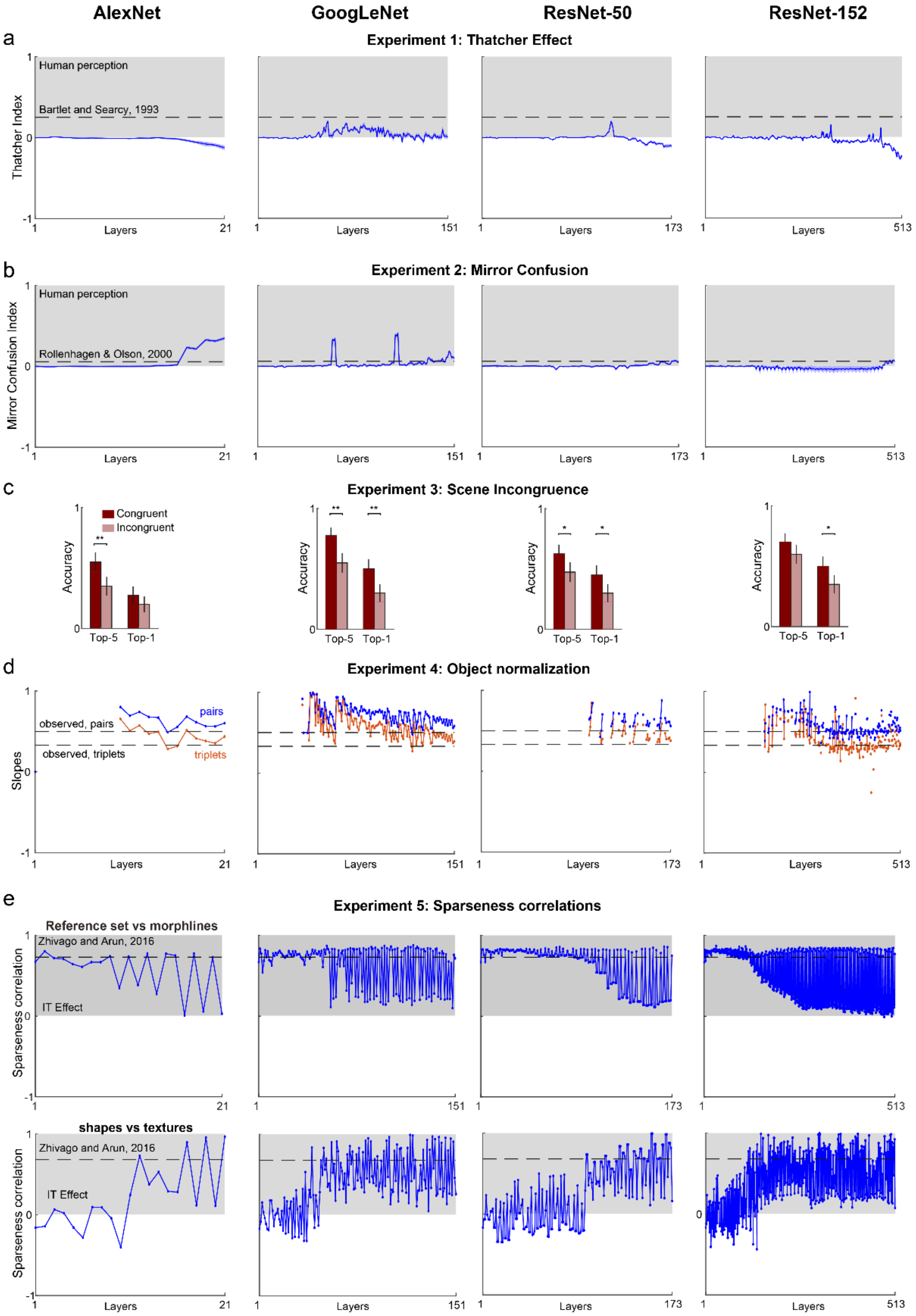

Figure S2. Experiments 1-5 with other feed-forward networks. Each column represents a deep network (from left to right: AlexNet, GoogLeNet, ResNet-50 and Reset-152). The grey region indicates human like performance.

a) Thatcher index across layers of the pretrained networks. Error bars indicate s.e.m across face pairs (n = 20). Dashed lines represent the Thatcher index from a previous study [1], measured on a different set of faces (see Methods).

b) Mirror Confusion Index (averaged across all objects) across layers for the pretrained deep networks. Error bars indicates s.e.m across stimuli (n = 50). Dashed lines represent the mirror confusion index estimated from monkey inferior temporal (IT) neurons in a previous study [2].
c) Accuracy of object classification by pretrained deep networks for congruent (*dark*) and incongruent (*light*) scenes, for top-5 accuracy (*left*) and top-1 accuracy (*right*). Error bars represent s.e.m across all scene pairs (n = 40). Asterisks indicate statistical significance computed using the Binomial probability of obtaining a value smaller than the incongruent scene correct counts given a Binomial distribution with the congruent scene accuracy (* is $p<0.05$,** is $p < 0.01$).
d) Normalization slope plotted across layers for two object displays (*blue*) and three-object displays (*red*) for pretrained deep networks. The dashed lines depict the slopes observed in monkey IT neurons using a different stimulus set [3].
e) *Top:* Correlation between sparseness on the reference set vs along morphlines across units of each layer in pretrained deep networks. The dashed line indicates the observed correlation in monkey IT neurons on the same set of stimuli. *Bottom:* Correlation between sparseness for textures and sparseness for shapes plotted across layers of pretrained deep networks. The dashed line indicates the observed correlation in monkey IT neurons on the same set of stimuli.

..

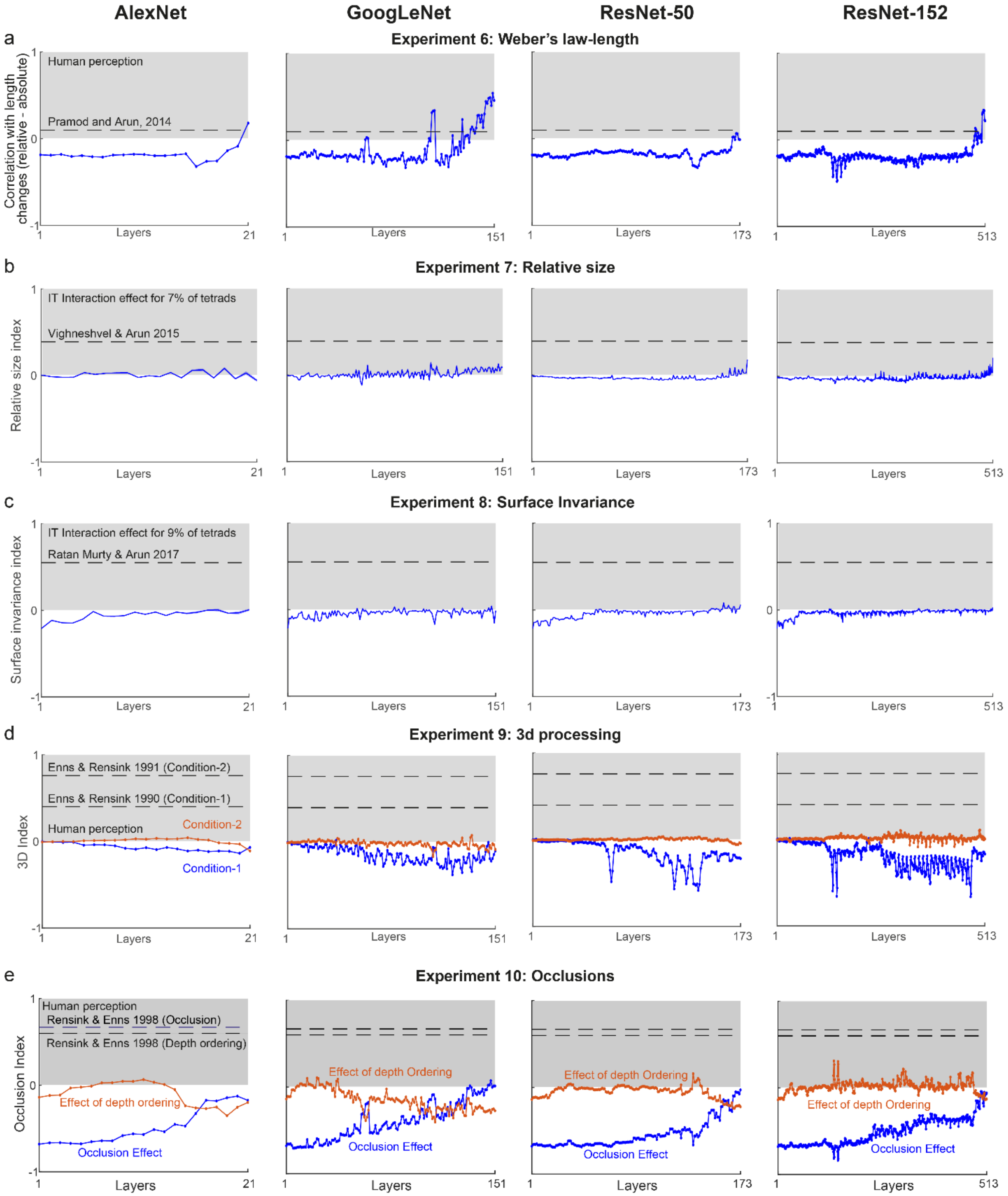

Figure S3. Results from Experiments 6-10 for other feedforward networks.
Each column represents a deep network (from left to right: AlexNet, GoogLeNet, ResNet-50 and ResNet-152).

a) To calculate a single quantity that measures adherence to Weber's law, we calculated the correlation between distances and relative changes in length and subtracted the correlation between distances with absolute changes in length (see

Methods). A positive difference indicates adherence to Weber's law (*gray region*). This difference in correlation is plotted across layers for line length in pretrained deep networks. The dashed line indicates the value observed during human performing visual search on the same stimuli.

b) Relative size index across units with interaction effects (averaged across top 7% tetrads , error bars representing s.e.m) across layers of pretrained deep networks. The dashed line shows the strength of the relative size index estimated from monkey IT neurons on the same set of stimuli [4].

c) Surface invariance index across units with interaction effects (averaged across top 9% pattern/surface tetrads, error bars representing s.e.m) across layers of pretrained deep networks. The dashed line depicts the surface invariance index estimated from monkey inferior temporal neurons on the same set of stimuli [5].

d) 3D processing index for pretrained deep networks across layers, for condition 1 and condition 2 (Figure 5A). Dashed lines represent the estimated human effect measured using visual search on the same stimuli.

e) Occlusion index for the occlusion and depth ordering effects for each layer of the pretrained deep networks. Dashed lines represent the effect size in humans measured using visual search on the same stimuli.

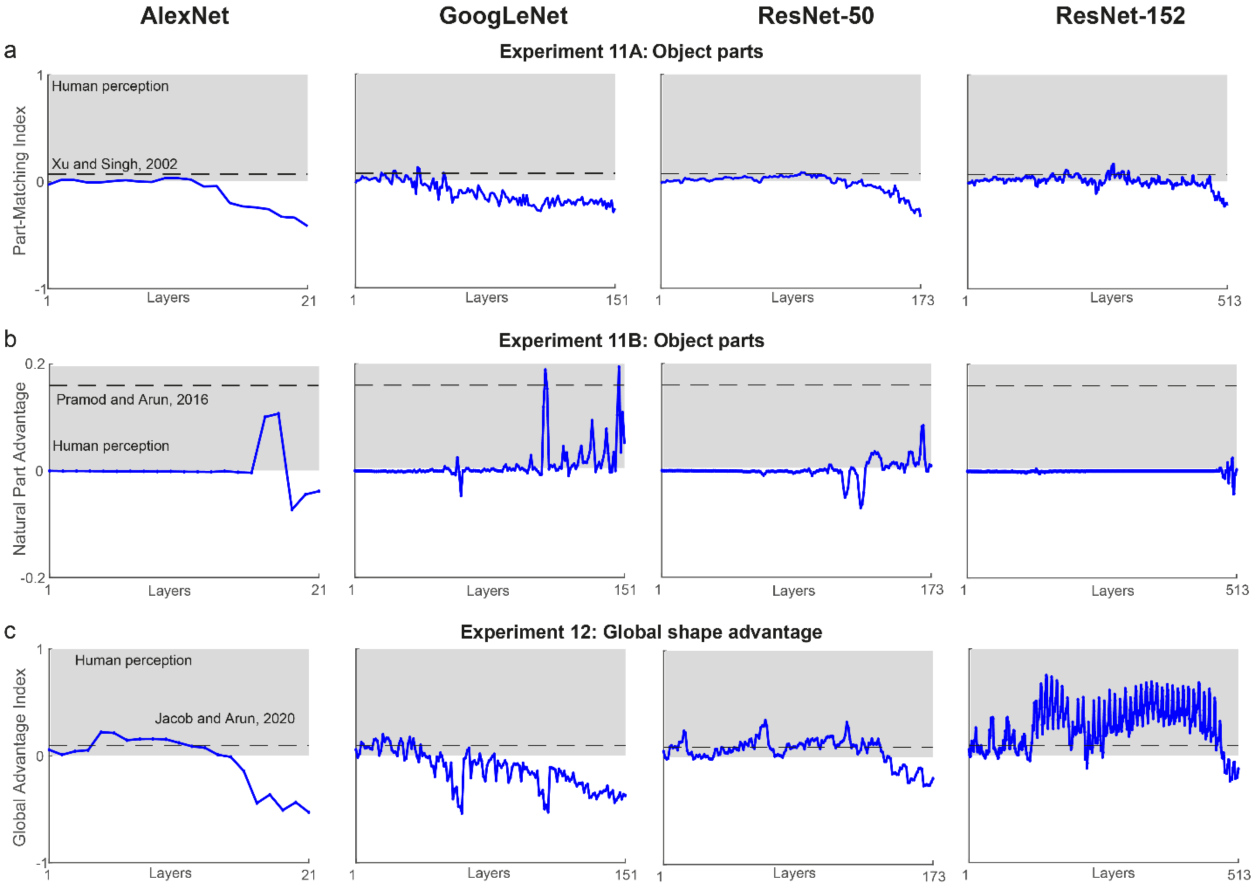

Figure S4. Results from Experiments 11-12 for other feedforward networks. Each column represents a deep network (from left to right: AlexNet, GoogLeNet, ResNet-50 and Reset-152).

a) Part processing index across layers of pretrained deep networks. The dashed line represents the effect size estimated from human visual search on the same stimuli [6].
b) Natural part advantage across layers of pretrained deep networks. The dashed line represents the effect size estimated from human visual search on the same stimuli [7].
c) Global Advantage index across layers of pretrained deep networks. The dashed line represents the effect size estimated from human visual search on the same stimuli [8].

SECTION S3. GENERALIZATION ACROSS DISTANCE METRICS

In the main text, we have presented results for all experiments using a Euclidean distance metric on deep neural network feature vectors. How sensitive are our results to the distance metric used? To address this issue, we repeated our analyses with three other distance metrics – city-block distance, Pearson linear correlation and Spearman rank correlation distances. We found that qualitatively similar results across distance metrics in nearly all experiments (Figure S5), except in a few cases where Pearson's correlation behaved differently from the other metrics, presumably because it is sensitive to outliers.

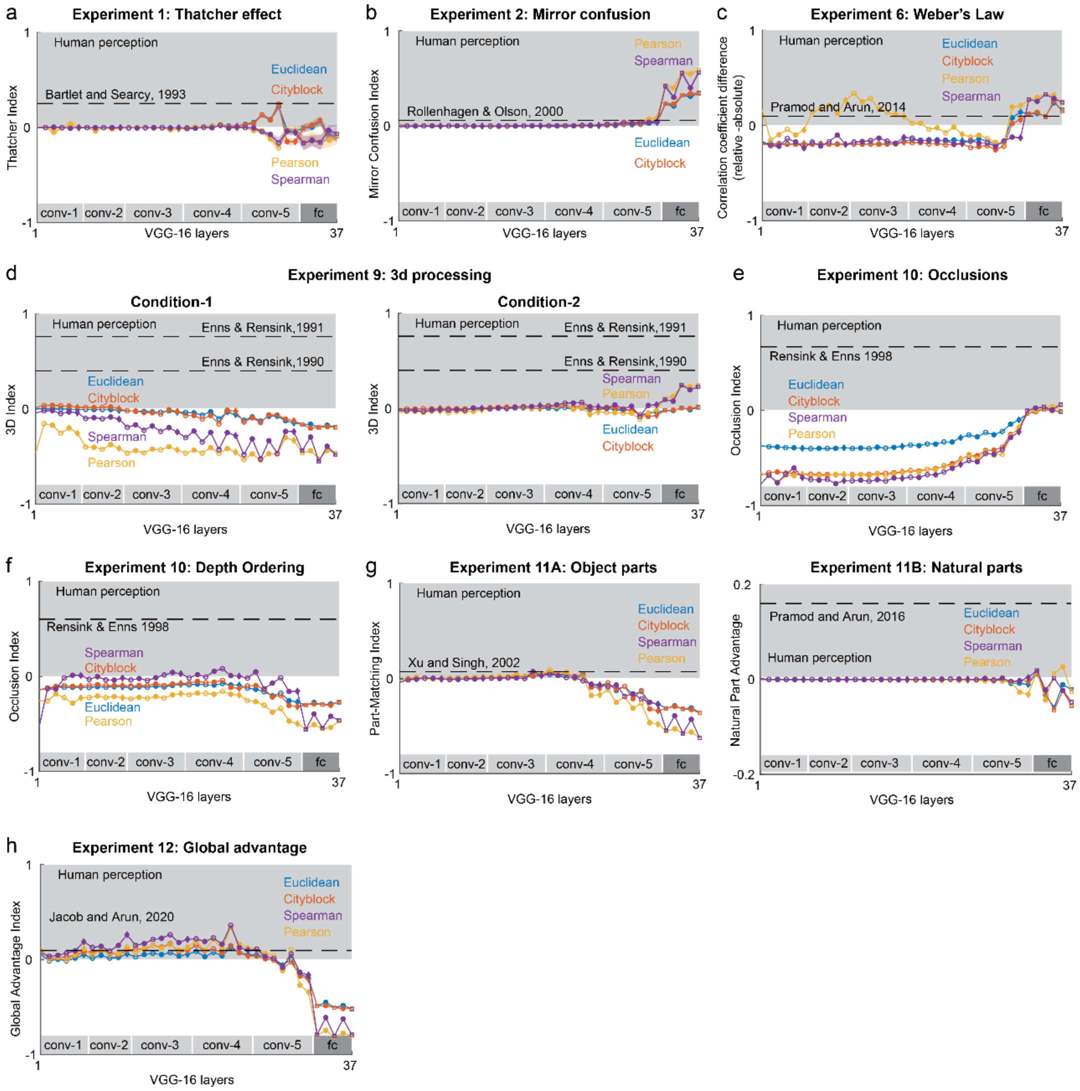

Figure S5. Generalization across different distance metrics. In each panel, the experiment-specific modulation index is shown for different distance metrics on VGG-16 feature vectors (Euclidean metric is *blue*, city-block in *red,* Pearson correlation distance in *yellow* and Spearman correlation distance in *purple)*.

a) Thatcher index across layers of the VGG network. Error bars indicate s.e.m across face pairs (n = 20). Dashed lines represent the Thatcher index from a previous study [1], measured on a different set of faces (see Methods).
b) Mirror Confusion Index (averaged across all objects) across layers for the pretrained VGG-16 network. Error bars indicates s.e.m across stimuli (n = 50). Dashed lines represent the mirror confusion index estimated from monkey inferior temporal (IT) neurons in a previous study [2].
c) Weber's law across layers of the VGG network. To calculate a single quantity that measures adherence to Weber's law, we calculated the correlation between distances and relative changes in length and subtracted the correlation between distances with absolute changes in length (see Methods). A positive difference indicates adherence to Weber's law (*gray region*). This difference in correlation is plotted across layers for line length in the VGG-16 networks. The dashed line indicates the value observed during human performing visual search on the same stimuli.
d) 3D processing index for the VGG-16 network across layers, for condition 1 and condition 2 (Figure 5A). Dashed lines represent the estimated human effect measured using visual search on the same stimuli.
e) Occlusion index for the occlusion effect across layers of the VGG-16 network. Dashed lines represent the effect size in humans measured using visual search on the same stimuli.
f) Occlusion index for the depth ordering effects across layers of VGG-16 network. Dashed lines represent the effect size in humans measured using visual search on the same stimuli.
g) Part processing index (*left*) and natural part advantage (*right*) across layers of the VGG-16 network. The dashed line represents the effect size estimated from human visual search on the same stimuli [6].
h) Global Advantage index across layers of the VGG-16 network. The dashed line represents the effect size estimated from human visual search on the same stimuli [8].

SECTION S4. LAYER-WISE ANALYSIS OF SCENE INCONGRUENCE

Unlike the other experiments, the scene incongruence experiment (Experiment 3) involves comparing classification accuracy, which does not elucidate how this effect emerges across layers. To investigate this possibility, we devised a novel analysis as detailed below.

The stimuli consisted of 40 pairs of images in which the same object image is pasted on to a congruent scene and an incongruent scene (see Methods, main text). These 40 objects were drawn from 36 unique ImageNet categories. We predicted that scene incongruence would manifest as a larger distance of incongruent scenes from the typical feature vector for each category. Accordingly, we first calculated a layer-wise centroid for each of the category by taking the average feature activation across 50 images in each category. We used images from test set of ImageNet for this analysis. We then calculated a scene incongruence index defined as $\frac{d_{incong} - d_{cong}}{d_{incong} + d_{cong}}$, where d_{incong} is the distance of the incongruent scene to the category centroid and d_{cong} is the distance of the congruent scene to the category centroid.

The results of this analysis are shown in Figure S6. The VGG-16 network showed an incongruence effect that increased only in the final fully connected layers (Figure S6A). By contrast, the randomly initialized VGG-16 network showed a positive effect that remained steady across layers, presumably reflecting some accidental property of the chosen scenes, or some similarity between object and scene features. We observed similar trends for both VGG-16 instances as well as across distance metrics (Figure S6A). Finally, we observed similar trends in the AlexNet (Figure S6B), GoogLeNet (Figure S6C), ResNet 50 (Figure S6D) and ResNet 152 (Figure S6E) although there were differences between networks in the exact progression across layers.

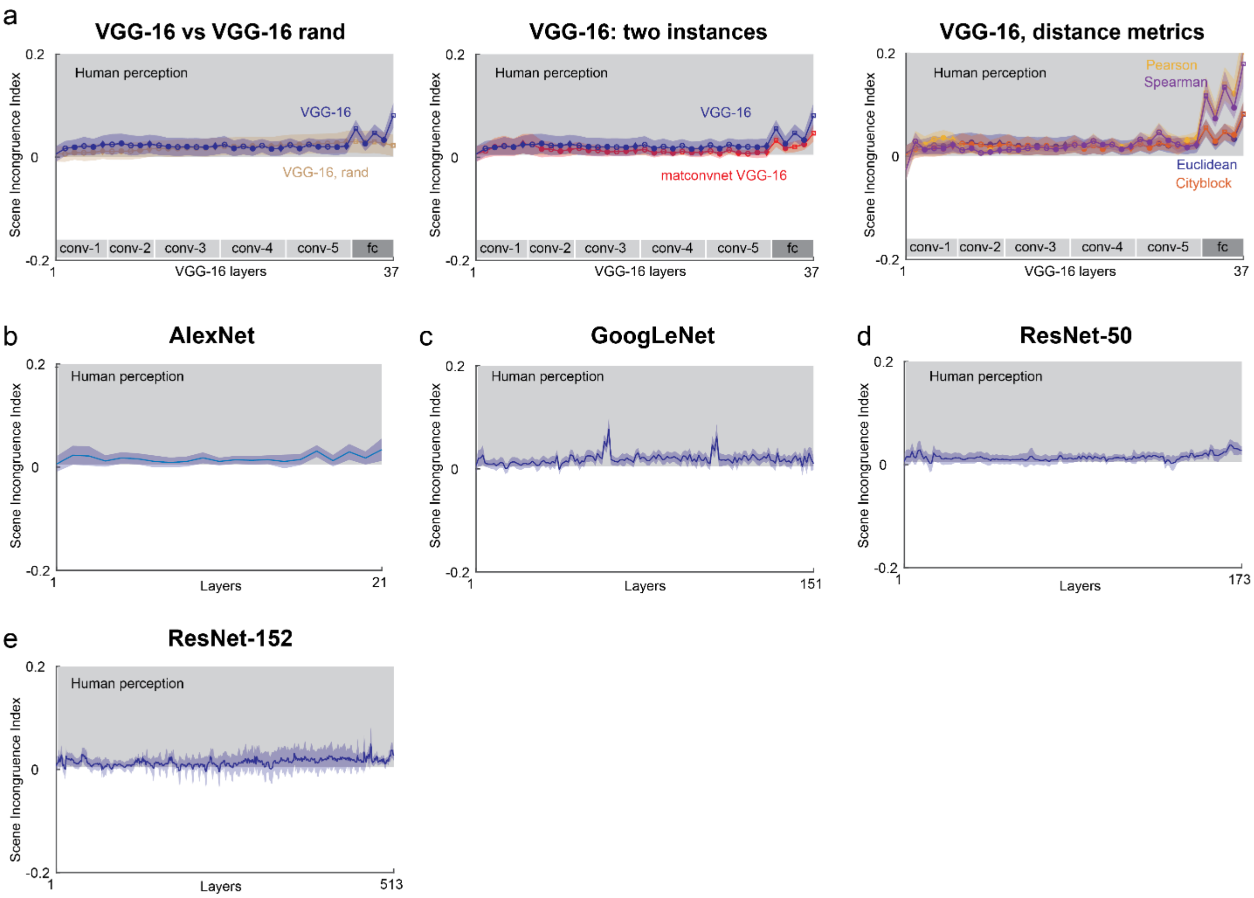

Figure S6. Progression of scene incongruence effect across layers.

a) Scene incongruence index across layers for VGG-16. *Left:* VGG-16 (*blue*) compared with a random network (*brown*). *Middle:* VGG-16 (*blue*) and the MatConvNet based VGG-16 (*red*). *Right:* Results with different distance metrics - Euclidean (*blue*), city-block distance (*red*), Pearson linear correlation distance (*yellow*) and Spearman rank correlation distance (*purple*). All other conventions are as before. Error bar represents s.e.m. across all scene pairs.
b) Scene incongruence index across layers for AlexNet. Error bar represents s.e.m. across all scene pairs.
c) Same as (B) but for GoogLeNet. Error bar represents s.e.m. across all scene pairs.
d) Same as (B) but for Resnet 50. Error bar represents s.e.m. across all scene pairs.
e) Same as (B) but for Resnet 152. Error bar represents s.e.m. across all scene pairs.

SECTION S5. SUPPLEMENTARY REFERENCES

1. Bartlett, J. C. & Searcy, J. Inversion and configuration of faces. *Cogn. Psychol.* **25**, 281–316 (1993).
2. Rollenhagen, J. E. & Olson, C. R. Mirror-Image Confusion in Single Neurons of the Macaque Inferotemporal Cortex. *Science (80-.).* **287**, 1506–1509 (2000).
3. Zoccolan, D., Cox, D. D. & DiCarlo, J. J. Multiple Object Response Normalization in Monkey Inferotemporal Cortex. *J. Neurosci.* **25**, 8150–8164 (2005).
4. Vighneshvel, T. & Arun, S. P. Coding of relative size in monkey inferotemporal cortex. *J. Neurophysiol.* **113**, 2173–2179 (2015).
5. Ratan Murty, N. A. & Arun, S. P. Seeing a straight line on a curved surface: decoupling of patterns from surfaces by single IT neurons. *J. Neurophysiol.* **117**, 104–116 (2017).
6. Xu, Y. & Singh, M. Early computation of part structure: Evidence from visual search. *Percept. Psychophys.* **64**, 1039–1054 (2002).
7. Pramod, R. T. & Arun, S. P. Object attributes combine additively in visual search. *J. Vis.* **16**, 8 (2016).
8. Jacob, G. & Arun, S. P. How the forest interacts with the trees: Multiscale shape integration explains global and local processing. *J. Vis.* **20**, 20 (2020).
9. LeCun, Y., Bengio, Y. & Hinton, G. Deep learning. *Nature* **521**, 436–44 (2015).
10. Liang, M. & Hu, X. Recurrent convolutional neural network for object recognition. *Proc. IEEE Comput. Soc. Conf. Comput. Vis. Pattern Recognit.* **07**-**12**-**June**, 3367–3375 (2015).
11. Deng, J. *et al.* ImageNet: A large-scale hierarchical image database. in *2009 IEEE Conference on Computer Vision and Pattern Recognition* **20**, 248–255 (IEEE, 2009).
12. Russakovsky, O. *et al.* ImageNet Large Scale Visual Recognition Challenge. *Int. J. Comput. Vis.* **115**, 211–252 (2014).
13. Krizhevsky, Alex, Ilya Sutskever, and G. E. H. ImageNet Classification with Deep Convolutional Neural Networks. *Adv. Neural Inf. Process. Syst. 25 (NIPS 2012)* 1–9 (2012).
14. Szegedy, C. *et al.* Going deeper with convolutions. in *2015 IEEE Conference on Computer Vision and Pattern Recognition (CVPR)* **91**, 1–9 (IEEE, 2015).
15. He, K., Zhang, X., Ren, S. & Sun, J. Deep Residual Learning for Image Recognition. in *2016 IEEE Conference on Computer Vision and Pattern Recognition (CVPR)* **45**, 770–778 (IEEE, 2016).
