# Supplementary material for: Qualitative similarities and differences in visual object representations between brains and deep networks
Source: Nat Commun. 2021 Mar 25;12:1872. doi: 10.1038/s41467-021-22078-3 (PMC7994307; doi:10.1038/s41467-021-22078-3)
Supplement: Supplementary file 3 — Reporting Summary [file 41467_2021_22078_MOESM3_ESM.pdf]

nature research

Corresponding author(s): SP Arun

Last updated by author(s): Jan 1, 2021

Reporting Summary

Nature Research wishes to improve the reproducibility of the work that we publish. This form provides structure for consistency and transparency in reporting. For further information on Nature Research policies, see our Editorial Policies and the Editorial Policy Checklist.

Statistics

For all statistical analyses, confirm that the following items are present in the figure legend, table legend, main text, or Methods section.

n/a	Confirmed	
☒	☐	The exact sample size (n) for each experimental group/condition, given as a discrete number and unit of measurement
☒	☐	A statement on whether measurements were taken from distinct samples or whether the same sample was measured repeatedly
☒	☐	The statistical test(s) used AND whether they are one- or two-sided<br>*Only common tests should be described solely by name; describe more complex techniques in the Methods section.*
☒	☐	A description of all covariates tested
☒	☐	A description of any assumptions or corrections, such as tests of normality and adjustment for multiple comparisons
☐	☒	A full description of the statistical parameters including central tendency (e.g. means) or other basic estimates (e.g. regression coefficient) AND variation (e.g. standard deviation) or associated estimates of uncertainty (e.g. confidence intervals)
☒	☐	For null hypothesis testing, the test statistic (e.g. F, t, r) with confidence intervals, effect sizes, degrees of freedom and P value noted<br>*Give P values as exact values whenever suitable.*
☒	☐	For Bayesian analysis, information on the choice of priors and Markov chain Monte Carlo settings
☒	☐	For hierarchical and complex designs, identification of the appropriate level for tests and full reporting of outcomes
☒	☐	Estimates of effect sizes (e.g. Cohen's d, Pearson's r), indicating how they were calculated

Our web collection on statistics for biologists contains articles on many of the points above.

Software and code

Policy information about availability of computer code

Data collection	All data generated in this study pertains to activations of neural networks to various images. This is publicly available at OSF (https://osf.io/35fmh/)
Data analysis	MatConvNet ver1.0 beta 24, Matlab 2019a, Active Shape Models Version 3.1 (http://www.milbo.users.sonic.net/stasm/), Manuscript Codes (https://osf.io/35fmh/)

For manuscripts utilizing custom algorithms or software that are central to the research but not yet described in published literature, software must be made available to editors and reviewers. We strongly encourage code deposition in a community repository (e.g. GitHub). See the Nature Research guidelines for submitting code & software for further information.

Data

Policy information about availability of data

All manuscripts must include a data availability statement. This statement should provide the following information, where applicable:

- Accession codes, unique identifiers, or web links for publicly available datasets
- A list of figures that have associated raw data
- A description of any restrictions on data availability

ImageNet database: http://www.image-net.org/, IISc Face Dataset: https://github.com/harish2006/IISCIFD

Field-specific reporting

Please select the one below that is the best fit for your research. If you are not sure, read the appropriate sections before making your selection.

☐ Life sciences ☒ Behavioural & social sciences ☐ Ecological, evolutionary & environmental sciences

For a reference copy of the document with all sections, see nature.com/documents/nr-reporting-summary-flat.pdf

Behavioural & social sciences study design

All studies must disclose on these points even when the disclosure is negative.

Study description	The aim of this study was to compare object representations in brains and deep neural networks using perceptual phenomena. We have formulated each perceptual phenomenon as a statement about distance relations. The primary data used for this study are images and all the analysis are on derived layer-wise feature activations obtained by passing these images through deep networks. The feature activations are quantitative.
Research sample	Not Applicable, as we have not done any behavioral or neural experiments and all the experiments are performed on deep networks.
Sampling strategy	Not Applicable, as we have not done any behavioral or neural experiments and all the experiments are performed on deep networks.
Data collection	Not Applicable, as we have not done any behavioral or neural experiments and all the experiments are performed on deep networks.
Timing	Not Applicable, as we have not done any behavioral or neural experiments and all the experiments are performed on deep networks.
Data exclusions	Not Applicable, as we have not done any behavioral or neural experiments and all the experiments are performed on deep networks.
Non-participation	Not Applicable, as we have not done any behavioral or neural experiments and all the experiments are performed on deep networks.
Randomization	Not Applicable, as we have not done any behavioral or neural experiments and all the experiments are performed on deep networks.

Reporting for specific materials, systems and methods

We require information from authors about some types of materials, experimental systems and methods used in many studies. Here, indicate whether each material, system or method listed is relevant to your study. If you are not sure if a list item applies to your research, read the appropriate section before selecting a response.

Materials & experimental systems

n/a	Involved in the study	
☒	☐	Antibodies
☒	☐	Eukaryotic cell lines
☒	☐	Palaeontology and archaeology
☒	☐	Animals and other organisms
☒	☐	Human research participants
☒	☐	Clinical data
☒	☐	Dual use research of concern

Methods

n/a	Involved in the study	
☒	☐	ChIP-seq
☒	☐	Flow cytometry
☒	☐	MRI-based neuroimaging
